# Environmental and occupational respiratory diseases – 1042. Influence of environmental endotoxin and allergen levels on prevalence of asthma and allergen sensitizations in urban and rural children in Guandong, China

**DOI:** 10.1186/1939-4551-6-S1-P41

**Published:** 2013-04-23

**Authors:** Mulin Feng, Liying Pan, Yan Chen, Jing Li

**Affiliations:** 1 Department of Allergy and Clinical Immunology, Guangzhou Institute of Respiratory Disease, the First Affiliated Hospital of Guangzhou Medical College, Guangzhou, China

## Background

To study the association between house dust endotoxin levels and respiratory symptoms and allergen sensitizations in children from the urban areas (Guangzhou) and rural areas (Conghua) of Guangdong province, China.

## Methods

Three hundred and seventeen children aged 13-14 years (Guangzhou 188, Conghua 129) with self-reported respiratory symptoms and 537 healthy children were selected from 854 school-children according to their response to ISAAC standardized questionnaire. These children attended the school infirmary again to complete a detailed questionnaire and medical examinations including skin prick test (SPT) to 8 common aeroallergens, lung function test, histamine bronchial provocation test and peripheral blood eosinophil counts. House dust samples were collected from living room and mattresses of 156 of these children with the agreement of their parents (Guangzhou 76, Conghua 80). The content of endotoxin in house dust was quantified using an endpoint chromogenic kinetic limulus amoebocyte lysate test. Associations between levels of house dust allergens and endotoxin and the provocation dose producing a 20% fall in forced expiratory volume in one second (PD_20_-FEV_1_) and allergen skin reactions were analysed by logistic regression.

## Results

The prevalence of wheeze symptom, asthma, nasal symptoms and allergic rhinitis in the children of Guangzhou were significantly higher (28.6%, 27.7%, 66%, 46.4%, respectively) than the children of Conghua (5%, 2.5%, 31%, 6.2%, respectively, p<0.01). Endotoxin concentrations in dust samples in homes of Conghua were significantly higher (1794 EU per square meter, 10.95 EU endotoxin/g dust) than those of Guangzhou (508.8 EU per square meter, 6.45 EU endotoxin/g dust, p<0.01). Logistic regression analyses showed a positive association between endotoxin levels and PD_20_-FEV_1_ (r=0.174, p<0.05), and a negative association (p<0.01) between endotoxin levels and the wheal sizes of skin response to Dermatophagoides pteronyssinus (Der. p) and Dermatophagoides farinae (Der. f).

## Conclusions

These findings suggest that exposure to higher levels of house dust endotoxin might contribute to the lower risk of allergic sensitization in children.

